# Socio-economic inequalities in overweight and obesity among women of reproductive age in Bangladesh: a decomposition approach

**DOI:** 10.1186/s12905-020-01135-x

**Published:** 2020-11-26

**Authors:** Emran Hasan, Moriam Khanam, Shafiun N. Shimul

**Affiliations:** 1 Department of Economics, Bangladesh University of Professionals (BUP), Dhaka, 1216 Bangladesh; 2 Institute of Health Economics, University of Dhaka, Dhaka, 1000 Bangladesh

**Keywords:** Overweight, Obesity, Inequality, Socio-economic factors, Decomposition, Bangladesh

## Abstract

**Background:**

Overweight and obesity of women is a growing concern all over the world. However, an understanding on the socio-economic inequalities in overweight and obesity of women received little attention, especially in the context of Bangladesh. Therefore, the objective of this study was to measure the inequality in overweight and obesity among women of reproductive age in Bangladesh as well as to explore the effect of various socio-economic factors on this inequality.

**Methods:**

This study used data from the Bangladesh Demographic and Health Survey 2014 which is a nationally representative data. The concentration index of overweight and obesity was applied to measure the extent of socio-economic inequality. Finally, the concertation index was decomposed in order to understand the contribution of different socio-economic variables in inequality in overweight and obesity of women.

**Results:**

This study included a total of 16,624 women of reproductive age. The study found that the prevalence of overweight was about 29% and the rate of obesity was approximately 11%. The value of concentration index for overweight and obesity was 0.37 (*p* < 0.001). This study also observed that about 52% inequality was explained by household’s wealth status followed by watching television (25%), husband/partner’s educational status (around 7%), women’s educational status (about 5%), place of residence (approximately 4%).

**Conclusions:**

This study found notable level of overweight and obesity among the women of Bangladesh. Various socio-economic factors like wealth status, education levels of women and partners, urban settings, women watching television predominantly contributed to the inequality in overweight and obesity among women of reproductive age. Therefore, the study suggests adopting necessary interventions targeting the women of higher socio-economic status to reduce the risk of life-threatening problems caused by overweight and obesity.

## Background

Overweight and obesity, a threat to the health and well-being of population, is a growing public health concern worldwide [1, 2]. In 2016, an estimated 1.9 billion adults were overweight, and 650 million were obese [3]. Though the problem of overweight and obesity is considered as a concern of developed countries, the recent statistics show that low and middle-income regions are facing a surge in the number of obese population [4]. Many low and middle-income countries are experiencing a double burden: rampant undernutrition and a rapid increase in non-communicable diseases due to obesity and overweight, especially in the urban settings [3]. Overweight and obesity influence the health and well-being adversely. People with overweight and obesity are likely to suffer from various life threatening non-communicable diseases such as hypertension, diabetes, cardiovascular diseases, cancer, osteoarthritis, etc.[4–6]. Globally, overweight and obesity combined accounted for about four million deaths and 120 million Disability Adjusted Life Years (DALYs) in 2015 [1, 7]. The effect becomes more devastating for women as it affects their health as well as the health of their children. About 40% women of age 18 and over were overweight and 15% of women were obese in 2016 [3]. Women with overweight and obesity are more likely to suffer from various pregnancy related complications such as gestational diabetes, gestational hypertension, pre-eclampsia and postpartum hemorrhage, instrumental delivery and surgical site infection to themselves and low birth weight, congenital malformation, preterm birth, large-for-gestational-age babies and perinatal death for the newborn [8–10]. Overweight and obesity of women at the time of pregnancy escalates the likelihood of childhood obesity which continues to adolescence and early adulthood, therefore, transmitting obesity to the next generation as well [11].

Bangladesh, a developing country, has also been experiencing a rise in overweight and obesity problems over the years. In the time span of 1999 to 2014, the prevalence of overweight women increased from 7.53 to 28.37% (almost four-fold) and the prevalence of obese women increased from 1.82 to 10.77% (about five-fold) [4]. This increasing prevalence is usually attributable to the recent economic progress along with demographic and nutritional transitions, urbanization, dietary and lifestyle changes [5, 12].

Many researches have been conducted to find out the predictors of overweight and obesity among women. These studies identified various determining factors including age, educational status of women, education of husband, marital status, wealth status, household size, number of children, watching television (TV) regional differentials that were associated with overweight and obesity of women [1, 4–6, 13]. However, an understanding on the socio-economic inequalities in the overweight and obesity received little attention, especially in the context of Bangladesh. Not knowing the extent of inequality along with its underlying reasons can misguide the policy priorities and thus resource allocation. Consequently, a targeted policy adoption will be difficult. Therefore, it is crucial to identify the extent of inequality of overweight and obesity across different socio-economic strata and determine the factors that contribute to the observed disparities. The study findings can help the policy makers design and prioritize various interventions over different groups to address the issue of overweight and obesity in Bangladesh and elsewhere with similar settings.

## Methods

### Study design and sampling procedure

This study used data from the Bangladesh Demographic and Health Survey (BDHS) 2014 which is a nationally representative cross-sectional survey. The survey collected data from women of reproductive age. Since 1993, the Ministry of Health and Family Welfare and National Institute of Population Research and Training (NIPORT) of Bangladesh have been conducting the DHS survey at three years interval. This survey was conducted from June 2014 to November 2014 which covers all the administrative divisions of Bangladesh. This survey interviewed a total of 17,863 ever-married women of the age group 15–49 years with a 98% response rate. The detailed methodology of sampling techniques, survey design, instruments, data collection methods, data reliability, validity, and quality control have been described elsewhere [14]. This study collected information on the socio-demographic characteristics of women, reproductive health, child health, nutritional status of mother and children, knowledge about sexually transmitted diseases. However, for this study, we have only considered women who have complete data on the anthropometric outcomes. We have excluded women who were pregnant during the survey as their weight would not be representative. After the application of the exclusion criteria, the sample size was 16,624.

### Outcome variable

Prevalence of overweight and obesity is the outcome variable of the current study. Measuring overweight and obesity using a single index is very challenging. However, Body Mass Index (BMI) is the most commonly used index to measure the overweight and obesity for adults. Like other studies, this study also used the information on respondents’ BMI to construct the overweight and obesity variable [4, 5, 12, 13, 15]. Respondents having 23 ≤ BMI < 27.5 was defined as overweight and BMI ≥ 27.5 was for obese as recommended by WHO for most Asian population [16]. We dropped the currently pregnant women while constructing the variable overweight and obesity due to higher BMI compared to usual. Finally, respondents having BMI ≥ 23 was categorized as overweight and obese. We categorized overweight and obesity as 1 (if respondents have BMI ≥ 23.5) and 0 otherwise.

### Explanatory variables

Previous empirical evidence suggested that there exists a statistically significant association between overweight and obesity and various socio-economic variables [1, 4, 5, 12, 13, 15, 17]. With an aim to estimate the socio-economic inequalities of overweight and obesity, the primary exposure variable was household’s wealth index which is often used to represent the household’s socio-economic status [18]. BDHS contains wealth index which was constructed using data on asset holdings and other household characteristics (i.e., roof material, floor material, refrigerators, availability of radio, television sets, mobile phones, ownership of land and livestock, sources of drinking water, water for washing and cooking, type of fuel for cooking, type of toilet facilities etc.) and using principal component analysis (PCA) following Filmer and Prichett [19]. Besides wealth index, this study incorporated respondents age, educational status, husband/partner’s educational status, place of residence, region, marital status, household size, number of children, caesarean section (ever had any caesarean section delivery), watching television and combination of women’s education and their husband/partner’s education following the previous established literature. Respondents’ age was categorized as 15–19, 20–29, 30–39, and 40–49 years. Educational status of respondents and respondents’ husband/partner was categorized as no education, primary, secondary, and higher education. Rural and urban were considered under place of residence while region was defined as Dhaka, Barisal, Chittagong, Rajshahi, Rangpur, Mymensingh, Khulna and Sylhet. Marital status was defined as ‘Yes’ if the respondent was married and ‘No’ if respondents were widowed/separated/divorced. Household size was categorized as 1–4, 5–6, and above 6 while number of children were defined as 1–2, 3–4 and 5 and above. Delivery by caesarean section was defined as ‘Yes’ if respondents ever had any caesarean section delivery and ‘No’ if otherwise. Watching television was categorized as ‘Yes’ if respondents watched television at least once a week and ‘No’ if otherwise. Finally, combination of women’s education and their husband/partner’s education variable was categorized following two steps as used in other studies [20, 21]. First, both women and their husband/partner’s education level were categorized as higher level of education if their education level was secondary or higher and lower level of education if their education level was primary or no education. Second, using these two categories, we constructed four combinations: (1) higher educated women with higher educated husband/partner, (2) higher educated women with lower educated husband/partner, (3) lower educated women with higher educated husband/partner, and (4) lower educated women with lower educated husband/partner.

### Statistical analysis

To accomplish the objective of this study, the analyses were performed in different stages using different methods—(1) concentration curve, (2) concentration index and (3) decomposition of concentration index.

Firstly, we employed the method of concentration curve with a view to analyzing the socio-economic inequality on the prevalence of overweight and obesity. Concentration curve was used to measure inequality by plotting the cumulative percentage/proportion/share of overweight and obese respondents against the cumulative percentage/proportion/share of respondents considering their socio-economic status [22]. Details on concentration curve is described elsewhere [23]. A 45^0^ line is generally used to represent the equality line while concentration curve lying above this line represents a higher concentration of socio-economic inequality amongst the bottom segments of respondents and vice-versa.

Secondly, concentration index summarized the graphical information (i.e. area between equality line and concentration curve) generated in concentration curve. The index was calculated using the following formula developed by Kawani [24], Jenkins [25] and Lerman and Yitzhaki [26].

$$CI = \frac{2}{\mu }{\text{cov}} \left( {h,r} \right);$$

where *CI* = concentration index; $\mu$ = (weighted) mean of underweight and obesity; *r* = fractional rank of the individual in the distribution of wealth index; *h* = overweight and obesity; cov = (weighted) covariance between *h* and *r*.

Properties of CI: (1) − 1 ≤ CI ≤ + 1; (2) CI = − 1 denotes socio-economic inequality is centered around the poorest quintile, (3) CI = + 1 denotes socio-economic inequalities are concentrated around the richest quintile, (4) CI = 0 denotes no significant socio-economic inequalities exists.

Finally, with a view to unearthing the contributions of differing socio-economic characteristics, we decomposed the concentration index calculated in the previous step. Following O’Donnell et al. (2008), we estimated the following regression (linear additive) equation at the first step [23].

$$Y = \upalpha + \Sigma_{{\text{K}}}\upbeta _{{\text{K}}} {\text{X}}_{{\text{K}}} +\upvarepsilon ;$$

where ${\upbeta }_{\mathrm{K}}$ = coefficient of the explanatory variables, ${\mathrm{X}}_{\mathrm{K}}$ = explanatory variables (selected socio-demographic variables), ε = (stochastic) error term.

The concentration index (CI) for overweight and obesity from the above-mentioned regression (Y) can be written as:

$${\text{CI}} = \Sigma_{{\text{K}}} {(\upbeta }_{{\text{K}}} {\overline{\text{X}}}_{{\text{K}}} /{\upmu )}{\text{C}}_{{\text{K}}} + {\text{GC}}_{\upvarepsilon } /\upmu$$

where CI = concentration index, µ = average of overweight and obesity (Y), ${\stackrel{-}{\mathrm{X}}}_{\mathrm{K}}$=average of Kth socio-economic variable(s), ${\mathrm{C}}_{\mathrm{K}}$ = concentration index for Kth socio-economic variable(s), = ${\mathrm{GC}}_{\upvarepsilon }$concentration index (generalized) for ε, ${(\mathrm{B}}_{\mathrm{K}}{\stackrel{-}{\mathrm{X}}}_{\mathrm{K}})$/µ = elasticity of overweight and obesity with respect to Kth socio-economic variable, ${\mathrm{GC}}_{\upvarepsilon }/\mu$= (residual component) denotes part of income induced inequality in overweight and obesity that cannot be explained by the explanatory variables. Results from the decomposition of concentration index were presented in elasticity, concentration index value, absolute contribution (same unit as the concentration index) and (adjusted) percentage (relative) contribution.

## Results

### Socio-demographic characteristics

This study included a total of 16,624 women of reproductive age for the survey year 2014 (Table 1). Most of the women (35.17%) belonged to age group 20–29 years. About 94% of women were married. The prevalence of overweight and obesity was about 29% and 11% respectively among the respondents. Most of the respondents (65.33%) lived in the rural areas and approximately 17% were from Dhaka region. Around 57% women had number of children 1–2 and most of respondents (42.46%) had small household size. Only 6.35% women ever had a caesarean delivery. More than half of the respondents had accessibility to electronic media (watch television at least once or every day in a week). Both the women and their partners were mostly secondary educated (about 37% and 29% respectively). Approximately, 83% of the respondents and their husband/partners were having primary or no education.

**Table 1:** Background characteristics of respondents

Variables	Variable frequencies (n = 16,624)	
	n	(%)
Age		
15–19	1667	10.03
20–29	5846	35.17
30–39	5180	31.16
40–49	3931	23.65
Marital status		
Widowed/divorced/separated	1014	6.10
Married	15,610	93.90
Nutritional status of women		
Underweight (BMI < 18.5)	3091	18.59
Normal (18.5 ≤ BMI < 23)	6922	41.64
Overweight (23 ≤ BMI < 27.5)	4751	28.58
Obese (BMI ≥ 27.5)	1860	11.19
No of children		
1–2	9419	56.66
3–4	5073	30.52
5 and above	2132	12.82
Household size		
1–4	7058	42.46
5–6	5611	33.75
Above 6	3955	23.79
Ever had any caesarean delivery		
No	15,568	93.65
Yes	1056	6.35
Watching TV (at least once or every day in a week)		
No	7998	48.11
Yes	8626	51.89
Wealth index		
Poorest	2999	18.04
Poorer	3102	18.66
Middle	3382	20.34
Richer	3534	21.26
Richest	3607	21.70
Educational status		
No education	4039	24.30
Primary	4875	29.33
Secondary	6153	37.01
Higher	1557	9.37
Husband's educational status		
No education	4766	28.67
Primary	4503	27.09
Secondary	4884	29.38
Higher	2471	14.86
Rural	10,860	65.33
Urban	5764	34.67
Region		
Barisal	1982	11.92
Chittagong	2639	15.87
Dhaka	2893	17.40
Khulna	2460	14.80
Rajshahi	2380	14.32
Rangpur	2389	14.37
Sylhet	1881	11.31
Combination of women’s education and their husband’s education		
Higher educated women with higher educated husband/partner	1495	8.99
Higher educated women with lower educated husband/partner	62	0.37
Lower educated women with higher educated husband/partner	1326	7.98
Lower educated women with lower educated husband/partner	13,741	82.66

### Prevalence of overweight and obesity and wealth status

**Fig. 1:**
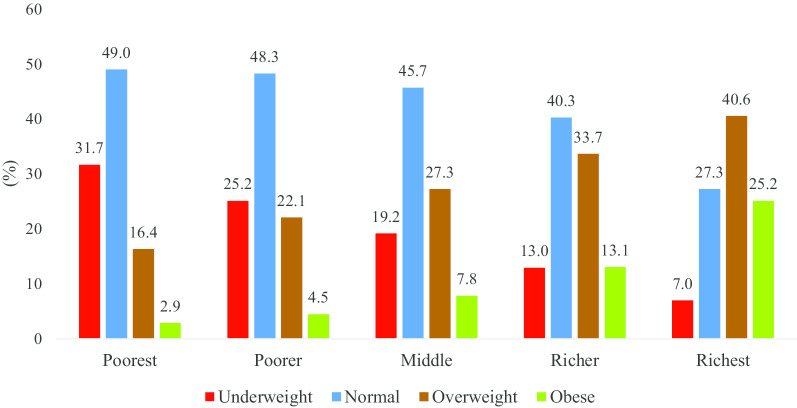
Overweight and obesity in Bangladesh accroding to wealth status

We found that the prevalence of overweight and obesity were more evident among the women belonging to upper quintiles (Fig. 1). Overweight and obese respondents were a mere 2.9% and 4.5% among the poorest and poorer quintiles respectively. On the other hand, the corresponding figures were 13.1% and 25.2% for the richer and richest quintiles respectively. Considering the polar opposite in the prevalence of overweight and obesity, underweight was more prevalent amongst the women from lower quintiles and vice-versa.

### Socio-economic inequalities in overweight and obesity: concentration curve and concentration index

**Fig. 2:**
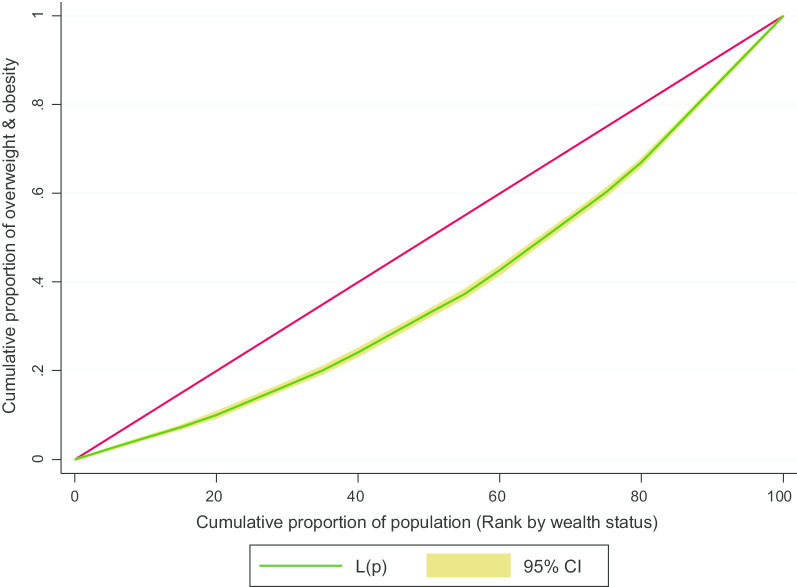
Concentration curve for overweight and obesity. Here, red line shows the line of equality and CI denotes confidence interval

We estimated the concentration curve and concentration index to represent the inequality in overweight and obesity for the survey year 2014. From the concentration curve, it was evident that the curve was lying below the line of equality (45^0^ line) representing socio-economic inequalities with higher concentration of overweight and obesity towards female from upper quintiles (middle, richer, richest) (Fig. 2). We found similar results from the estimated concentration index as well. The value of concentration index for overweight and obesity was 0.37 (*p* < 0.001) in 2014—representing a pro-rich wealth-based socio-economic inequality.

### Decomposition of concentration index for overweight and obesity

**Fig. 3:**
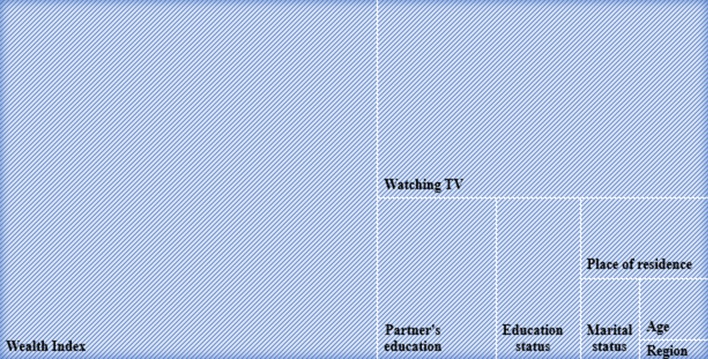
Percentage contribution of socio-economic factors in concentration index

We decomposed the concentration index to explore the contribution of different socio-economic variables for inequalities in overweight and obesity. Decomposition results (i.e. elasticity, concentration index, absolute contribution, and percent contribution) are presented in Table 2. Our results suggested that women from Khulna, Rangpur, Rajshahi and Sylhet, women and their husband/partners’ having little or primary education, women from lower (poorer and middle) quintiles of households, with the higher number of children (3–4, 5 and above), belonging to households with 5–6 members were concentrated amongst the bottom segment of the population on the question of inequalities in overweight and obesity. Women living in urban areas especially from Dhaka and Chittagong, belonging to upper (middle, richer and richest) quintiles of households, watching television, having a caesarean delivery, being married, women and their husband/partners’ having more than primary education and women from household size seven and above were more concentrated on the upper segment of the population in terms of inequalities in overweight and obesity. Household wealth status, watching television, husband/partners educational status, place of residence, women’s educational status had the highest contribution to the inequality in overweight and obesity (Fig. 3). About 52% inequality was explained by the household’s wealth status followed by watching television (about 25%). Women’s husband/partner’s educational status explained around 7%. Furthermore, women’s educational status had a significant contribution to overweight and obesity-related inequality (slightly over 5%). About 4% of the inequalities in overweight and obesity was explained by place of residence followed by marital status (1.84%), age (1.614%) and region of residence (0.506%).

**Table 2:** Contribution of socio-demographic characteristics based on the decomposition of concentration index (CI) for overweight and obesity in Bangladesh (2014)

Socio-economic variables	Elasticity	Concentration index (CI)	Absolute contribution	Percentage contribution
Age				
15–19 (ref)				
20–29	0.286	0.004	0.001	0.285
30–39	0.437	0.006	0.003	0.766
Above 40	0.317	0.007	0.002	0.563
Sub-total			0.006	1.614
Marital status				
Married	0.356	0.019	0.007	1.840
Widowed/divorced/separated (ref)				
Sub-total			0.007	1.840
No of children				
1–2 (ref)				
3–4	− 0.013	− 0.075	0.001	0.270
5 and above	− 0.026	− 0.097	0.003	0.696
Sub-total			0.004	0.966
Household size				
1–4 (ref)				
5–6	− 0.019	− 0.062	0.001	0.315
7 and above	− 0.037	0.061	− 0.002	− 0.606
Sub-total			− 0.001	− 0.291
Cesarean section delivery				
Yes	0.007	0.087	0.001	0.166
No (ref)				
Sub-total			0.001	0.166
Watching TV				
Yes	0.135	0.674	0.091	24.866
No (ref)				
Sub-total			0.091	24.866
Wealth index				
Poorest (ref)				
Poorer	0.058	− 0.333	− 0.019	− 5.246
Middle	0.088	− 0.040	− 0.003	− 0.948
Richer	0.160	0.308	0.049	13.448
Richest	0.244	0.669	0.163	44.400
Sub-total			0.190	51.654
Educational status				
No education (ref)				
Primary	0.042	− 0.153	− 0.006	− 1.730
Secondary	0.091	0.252	0.023	6.237
Higher	0.015	0.178	0.003	0.705
Sub-total			0.020	5.212
Husband’s education level				
No education (ref)				
Primary	0.035	− 0.117	− 0.004	− 1.123
Secondary	0.065	0.222	0.014	3.939
Higher	0.060	0.274	0.016	4.462
Sub-total			0.026	7.278
Place of residence				
Rural (ref)				
Urban	0.031	0.463	0.014	3.907
Sub-total			0.014	3.907
Region				
Dhaka	0.004	0.173	0.001	0.197
Chittagong	0.021	0.079	0.002	0.450
Khulna	0.025	− 0.005	0.000	− 0.034
Rangpur	0.000	− 0.125	0.000	0.008
Rajshahi	0.013	− 0.061	− 0.001	− 0.219
Sylhet	− 0.013	− 0.029	0.000	0.104
Barisal (ref)				
Sub-total			0.002	0.506

The study also attempted to investigate the contribution of the combination of women and their husband/partner’s educational attainment on the inequalities of overweight and obesity through decomposing the concentration index. The findings suggested that higher educated women with lower educated husband/partner and lower educated women with higher educated husband/partner were more concentrated in the upper segment of the population in terms of the inequalities in overweight and obesity (Table 3). Despite the percentage contribution of these two categories in the inequalities in overweight and obesity was very small, still lower educated women having higher educated husband/partner contributed higher (0.394%) compared to higher educated women having lower educated partner/husband (0.001%). On the other hand, both women and their husband/partner having lower educational attainment were concentrated more in the bottom segment of the population on the question of inequalities in the overweight and obesity. The percentage contribution of this category in the inequalities in overweight and obesity was more than 12%.

**Table 3:** Contribution of the combination of women’s education and their husband/partner’s education based on the decomposition of concentration index (CI) for overweight and obesity in Bangladesh (2014)

Combination of women and their husband/partner’s education	Elasticity	Concertation index (CI)	Absolute contribution	Percentage contribution
Higher educated women with higher educated husband/partner (ref)				
Higher educated women with lower educated husband/partner	0.001	0.002	0.000	0.001
Lower educated women with higher educated husband/partner	0.011	0.128	0.001	0.394
Lower educated women with lower educated husband/partner	− 0.149	− 0.306	0.046	12.425
Sub-total			0.047	12.820

Decomposition of concentration index (CI) for overweight and obesity was conducted adjusted for age, marital status, no of children, household size, cesarean section delivery, watching TV, wealth index, Place of residence and region of residence. Women’s education and their husband/partner’s educational status were excluded from this model due to the fact that inclusion of these two variables along with the variable—combination of women and their husband/partner’s educational status might create collinearity problem

## Discussion

Using BDHS data 2014, this study aimed to assess the socio-economic inequalities in overweight and obesity among women of the reproductive age in Bangladesh. We quantified the contribution of each of socio-demographic factors to the inequalities by employing CI method and decomposition of the CI. This study found that the prevalence of overweight was 20% while the rate of obesity was 4.5%. Therefore, overweight and obesity remains a significant concern in Bangladesh. This study found that women belonging to upper (middle, richer and richest) quintiles of socio-economic status (SES), living in urban areas, watching television, married, women and their husband/partners’ having more than primary education were more concentrated on the upper segment of the population in terms of inequalities in overweight and obesity.

We found that prevalence of overweight and obesity were more concentrated towards the women from the upper wealth quintiles. Previous studies carried out with Bangladesh, Nigeria, Ethiopia settings also observed similar findings, as it is widely perceived that overweight and obesity is more pro-rich in the developing country settings while it is pro-poor centric in the developed ones [1, 4, 12]. This may happen due to the fact that women from wealthier families in lower income countries have greater access to food, have lower level of physical activity and thereby at a greater risk of overweight and obesity [4]. This study also found that educated women were proportionally more vulnerable to overweight and obesity. Other studies from different developing countries including Bangladesh also found similar result [1, 4, 27]. The educated women are more likely to do a sedentary job which require less physical activity and therefore, have greater risk of weight gain [4, 28]. A study showed that Bangladeshi women has the highest level of physical inactivity when compared to other South Asian women [29]. In addition, lack of access to park and well-managed sidewalks, availability of household helping hands can explain this lower level of physical activity. Moreover, the increased inequality in overweight and obesity among women with higher education and higher wealth index might be related to changing nutritional and lifestyle pattern, consumption of high-calorie foods [1]. On the contrary, the findings from the study conducted in the North-West of Iran suggested that respondents’ having higher level of education were less vulnerable to obesity [30]. This was attributed to the fact that educated respondents might possess adequate knowledge about the importance of healthy lifestyle and balanced diets intake. Our findings showed that women whose husbands had higher educational status were more prone to overweight and obesity and the result was consistent with other studies in Bangladesh [4]. This might happen due to the fact that educated husbands are more likely to earn more and to do sedentary jobs. In addition, eating out is also likely to be more in this segment with increased consumptions of junk or fast foods resulting in unhealthy and excessive weight.

We also found that women watching TV at least once or every day in a week were suffering more from overweight and obesity compared to women who did not watch TV. Studies from Bangladesh and other countries also show that watching TV is strongly correlated with overweight and obesity among women [5, 17]. While watching TV has some educational and recreational benefits, this is also good proxy for spending time sedentarily. In addition, due to cultural reasons, many women remain out of labor force with plenty of leisure time and they spend enormous amount of time watching television [5]. Furthermore, while watching TV, consumption of different obesogenic foods such as chips, fried potatoes, sausages, bologna, pizzas, hamburgers, butter, nuts etc. can also contribute to weight gain.

This study found that urban women were more likely to suffer from overweight and obesity compared to the women from the lower ladder. The finding is in line with many other studies [1, 4]. The possible reason for this disparity in overweight and obesity between urban and rural women might be in the rural areas, women are engaged in various physical activities like agricultural and other activities, therefore, unlikely to gain as much weight as the urban women [1]. On the other hand, women in urban areas are more exposed to sedentary life, reduced physical activity, eating packed and high calorie food [15]. Therefore, women in urban areas require urgent interventions such as changes in bad eating habits (like fast foods) and sedentary lifestyles to reduce the risk of overweight and obesity.

Our study revealed that married women were more susceptible to overweight and obesity. Other studies in Bangladesh also found that women who were not living with their husbands or separated had lower probability of being overweight and obese compared to married women [15]. A significant number of women in Bangladesh get married while they were child, and so did not complete their education resulting no participation in the labor force, and this group spends enormous amount of time at home with very limited physical activities. Therefore, they are at greater risks of being overweight and obese.

This study also found that higher educated women with lower educated husband were more concentrated in the upper segment of the population in terms of the inequalities in overweight and obesity. A study in Japan found that educational difference between spouses when educated women were married to less educated partner resulted in a greater risk of overweight and obesity among women [20]. This might be due to the fact that unhealthy lifestyle of less educated partner may influence the risks of overweight and obesity among women. Moreover, our study found that lower educated women with higher educated husband were more concentrated in the upper quintiles of the population on the question of the inequalities in overweight and obesity. This reasoning behind this might be that lower educated women who had higher educated husband are in a disadvantageous position in terms of labor force participation. Therefore, they remain mostly at home and engaged in household activities requiring less physical movements.

Even through this study has uncovered some key questions of inequality in obesity and overweight in Bangladesh, it has a few limitations that is worth mentioning: (1) as the nature of the dataset was cross-sectional, we could not extract any direct causal relationship among the variables studied, and (2) we could not control for some potential confounders like energy intake, smoking habit, physical activity of women, availability of sports equipment, dietary pattern of the respondents as BDHS-2014 did not collect detailed data on these variables. Despite some potential limitations, the core strength of our study was the use of nationally representative data with robust estimation methods. Hence, we can generalize these findings to the whole population.

## Conclusions

The current study found alarming level of overweight and obesity among the women of Bangladesh. The study also found that various socio-economic factors like wealth status, education levels of women and partners, urban settings, women watching television frequently contributed most to the inequality of overweight and obesity among women of reproductive ages. Married women also had greater risk of having higher BMI status. Therefore, there is an urgent need to take interventions targeting the women from higher socio-economic status to reduce the risk of life-threatening problems caused by overweight and obesity. Furthermore, policy makers should formulate and implement various awareness raising initiatives regarding the adverse impact of overweight and obesity, especially in the urban setting.

## Data Availability

The dataset (BDHS 2014) used in this study is publicly available in the DHS website (https://dhsprogram.com/data/dataset/Bangladesh_Standard-DHS_2014.cfm?flag=0).

## Abbreviations

DALYs: Disability Adjusted Life Years
TV: Television
BDHS: Bangladesh Demographic and Health Survey
NIPORT: National Institute of Population Research and Training, DHS: Demographic and Health Survey
BMI: Body Mass Index
PCA: Principal Component Analysis
WHO: World Health Organization
CI: Concentration Index
CI: Confidence Interval
SES: Socio-economic status
